
JOURNAL INFORMATION
==============================
NLM Title Abbreviation: Inter Econ
Journal ID: Inter Econ
NLM Journal ID: 101086399

Inter Economics

ISSN: 0020-5346

ARTICLE INFORMATION
==============================
PMCID: PMC8021641
PMID: 33840830
DOI: 10.1007/s10272-021-0965-x
Article ID: 965
Article version: 1
Subjects: Letter from America

Making the Most of Their Shot: The American Rescue Plan Package

Posen Adam S. posena@piie.com

grid.487624.b 0000000121495725 Peterson Institute for International Economics, 1750 Massachusetts Avenue, NW, 20036-1903 Washington, DC, USA
Electronic publication date: 2021 Apr 6
Print publication date: 2021
Volume: 56
Issue: 2
First page: 127
Last page: 128
Copyright: © The Author(s) 2021
License: Open Access: This article is distributed under the terms of the Creative Commons Attribution 4.0 International License (https://creativecommons.org/licenses/by/4.0/).
Open Access funding provided by ZBW — Leibniz Information Centre for Economics.
License URL: https://creativecommons.org/licenses/by/4.0/

issue-copyright-statement: © The Author(s) 2021

==============================

The Biden Administration and the very narrow Democratic party majorities in Congress see the country as facing three existential threats simultaneously: the COVID-19 pandemic, climate change, and the attack on American elections and the constitution from parts of the Republican party. As a result, they believe that they have a narrow window ahead of the November 2022 midterm elections to make major policy moves that deliver on electoral promises. They hope to demonstrate that democratic and Democratic government are desirable for a clear majority of voters in the US – a large majority is needed for Democrats to secure a working majority in government due to the configuration of the US electoral system and voter suppression efforts by Republicans.

Therefore, it should not come as a surprise that the American Recovery Plan (ARP) legislation passed along a strict party line vote and, more importantly, included the full $1.9 trillion of additional spending initially proposed. This package comes on top of the $900 billion pandemic relief bill Congress passed in September that included stimulus payments of as much as $600 for many individuals, enhanced unemployment benefits and provided more support for small businesses. The element of the ARP that attracts the most attention is the top up of the stimulus payments to individuals earning less than $75,000 to $2,000 each. On paper, this is deficit spending of roughly 14% of GDP to be paid out in 2021.

Broadly speaking, that is an exaggeration of the cumulative fiscal spending, but not one that changes the implications very much. Both packages combine spending directly dedicated to working the problem of the pandemic in the limited US welfare state and unemployment system, with actual stimulus payments. Roughly $1.1 trillion of the ARP package fits in that first category, meaning direct funding for vaccine distribution, public health measures, state and local government capacities, and an extension of unemployment benefits. These are essentially self-liquidating measures, meaning that when vaccines are distributed, state governments balance their budgets, or workers return to employment, the spending will automatically stop. So, the actual expenditure is likely to be at least $200 billion less. The state and local governments in particular are in better financial shape on average than expected, and they will bank much of the transfer from the federal government. Another $350 to $400 billion of projected spending will be disbursed over more than just the next nine months.

But this point is in some sense a moot point as it still leaves 10% of GDP in deficit-financed stimulus for the US economy in the remainder of calendar year 2021, perhaps even 2% more than that depending upon how you allocate the December money. This is why Olivier Blanchard and Lawrence Summers have both expressed their concerns about overheating as a result of the ARP. Official estimates of the pre-ARP output gap for the US economy (for example, of 4% of GDP by the Congressional Budget Office) are biased downwards. That bias, however, is not enough to offset the mismatch between a realistic output gap (of say 6% of GDP) and the stimulus of 10% to 12%.

Does the significance of this mismatch depend upon the multiplier of the various fiscal components? Some economists, including those in the Biden Administration advocating for the plan, contend that the multiplier will be quite low. If that is the case, however, it is reasonable to question how much of this spending beyond targeting the pandemic problem, particularly the stimulus checks, is urgently needed. But even allowing for low multipliers on all the directly pandemic-related spending and state and local government transfers does not remove the issue. Much of the recession in the US economy is due to the coronavirus and fears of it (not the lockdown), so when, hopefully, the vaccines are widely accepted and work, there will be a growth rebound even without stimulus.

The available evidence suggests that a considerable amount of pent-up demand is poised to provide a strong boost to spending once restrictions are lifted and people view it as safe to interact with others again. Personal saving in 2020 was about $1.5 trillion above what one might have expected based on pre-pandemic behavior – the equivalent of 7% of GDP. We should expect "excess saving" to rise even further before it peaks, boosted in part by income associated with the additional fiscal measures described.

As my colleague Karen Dynan has pointed out, even at the bottom end of the income distribution, where unemployment is now concentrated, there is little evidence that the loss of earnings has translated into widespread financial distress that will constrain spending. For example, data from the Opportunity Insights Economic Tracker suggest that consumer spending in low-income zip codes has remained close to pre-pandemic levels. The vast majority of tenants are still current on their rents, and bankruptcy filings have remained low. This outcome does not mean that lower-income households have been insulated from the economic fallout associated with the pandemic. Rather, it should be interpreted as a near-term victory for the aggressive fiscal and monetary support put in place last spring.

I would go even further with regards to savings behavior. After the Great Depression, we saw the US personal savings rate rise on a lasting basis for young people, after some transitional dynamics. We saw a similar lasting rise in the household savings rate for the US after the 2008-10 financial crisis. The rate was 3.5% prior to 2008; it shot up during the crisis of course, and then stabilized around 7.5% after 2012. We should expect something similar in coming years for young people who have now seen their second exogenous mass unemployment shock in just over a decade, meaning the savings rate would stabilize at 9% to 10% – but only after a few years of transition dynamics.

There is some push back on the pent-up savings leading to the spending forecast. Durable goods and residential construction spending were already strong over recent months, as well as auto sales. Meanwhile, some observers point out that consumers cannot have too much pent-up demand for many services, whether haircuts or restaurant meals, which cannot be accumulated. This overlooks an important sector, health care, which makes up at least 18% of total US GDP. There is ample evidence that due to fear over the COVID-19 virus and shifts in medical availability, many 'elective' health procedures were foregone by consumers in 2020. These elective exams, operations and treatments run the gamut from cosmetic surgery to cancer follow-ups. As a result, these have likely truly pent-up demand that will be expressed in coming months. A similar argument on a much smaller share of GDP can be made for higher education.

So, there will almost certainly be overheating and a historic boom in US GDP growth. Forecasts of 8% real year-over-year GDP growth are plausible and common, and US real GDP is projected to surpass its pre-pandemic high in the middle of this year. But what does that overheating actually mean? Even those concerned about excess spending are rightly reluctant to forecast runaway inflation. As Joseph Gagnon argues, the proper historical parallel for this period is probably the 1950s following the visibly finite temporary spending on the Korean War, starting from a small but extant output gap and unemployment. This is not the inflationary 1970s, where demand above potential was sustained long after unemployment fell, and government programs showed no reason to be deemed temporary. While the Federal Reserve could turn temporary inflation into a persistent problem, this is far from likely in a world where we have had trouble achieving even 2% target inflation for years.

Adam S. Posen, Peterson Institute for International Economics, Washington DC, USA.

